# Prevalence of and factors associated with low Back pain, thoracic spine pain and neck pain in Bashkortostan, Russia: the Ural Eye and Medical Study

**DOI:** 10.1186/s12891-020-3080-4

**Published:** 2020-02-01

**Authors:** Mukharram M. Bikbov, Gyulli M. Kazakbaeva, Rinat M. Zainullin, Venera F. Salavatova, Timur R. Gilmanshin, Inga I. Arslangareeva, Nikolai A. Nikitin, Svetlana R. Mukhamadieva, Dilya F. Yakupova, Songhomitra Panda-Jonas, Renat I. Khikmatullin, Said K. Aminev, Ildar F. Nuriev, Artur F. Zaynetdinov, Yulia V. Uzianbaeva, Jost B. Jonas

**Affiliations:** 1 Ufa Eye Research Institute, 90 Pushkin Street, Ufa, 450077 Bashkortostan Russia; 2 Department of Ophthalmology, Medical Faculty Mannheim of the Ruprecht-Karls-University of Heidelberg, Mannheim, Germany

**Keywords:** Back pain, Neck pain, Population-based study, Ural eye and medical study

## Abstract

**Background:**

Back pain and neck pain are leading causes of the burden of disease worldwide, while information about their prevalence in Russia is missing.

**Methods:**

The population-based Ural Eye and Medical Study was conducted in a rural and urban region in Bashkortostan/Russia. As part of a detailed systematic examination, we assessed the prevalence of low back pain, thoracic spine pain and neck pain in an interview with standardized questions in 5397 study participants (mean age:58.6 ± 10.6 years;range:40–94 years).

**Results:**

The mean prevalence of low back pain, thoracic spine pain and neck pain was 2912/5397 (54.0%;95% confidence interval (CI):52.6,55.3), 1271/5397 (23.6%;95%CI:22.4,24.7), and 1570/5397 (29.1%;95%CI:27.9,30.3), respectively. A higher prevalence of low back pain was associated with females (*P* = 0.04;odds ratio (OR):1.14;95%CI:1.004,1.30), younger age (*P* < 0.001;OR:0.99;95%CI:0.98,0.99), higher body mass index (*P* = 0.002;OR:1.02;95%CI:1.01,1.03), lower frequency of vigorous activities during leisure time (*P* = 0.001;OR:0.79;95%CI:0.69,0.90), more time spent sitting and reclining (*P* = 0.03;OR:1.00;95%CI:1.00,1.00), higher serum concentration of high-density lipoproteins (*P* = 0.004;OR:1.10;95%CI:1.03,1.18), higher prothrombin index (*P* = 0.003;OR:1.01;95%CI:1.003,1.01), higher prevalence of a history of cardiovascular disease (*P* = 0.004;OR:1.23;95%CI:1.07,1.42), falls (*P* < 0.004;OR:1.71;95%CI:1.45,2.00), bone fractures (*P* = 0.01;OR:1.18;95% CI:1.03,1.34), unconsciousness (*P* < 0.001;OR:1.78;95%CI:1.40,2.25), osteoarthritis (*P* < 0.001;OR:2.76;95%CI:2.34,3.26), iron-deficiency anemia (*P* < 0.001;OR:1.87;95%CI:1.41,2.50), and thyroid disorder (*P* = 0.004;OR:1.37;95%CI:1.10,1.70), fewer days of vegetable intake (*P* < 0.001;OR:0.89;95%CI:0.85,0.93), smaller amounts of salt intake (*P* = 0.008;OR:0.97;95%CI:0.94,0.99), higher anxiety score (*P* < 0.001;OR:1.05;95%CI:1.03,1.06), and in women, history of menopause (*P* = 0.02;OR:1.36;95%CI:1.05,1.75). The prevalence of thoracic spine pain and neck pain showed similar associations.

**Conclusions:**

In a Russian population, the prevalence of low back pain, thoracic spine pain and neck pain (54.0, 23.6 and 29.1%, respectively) were correlated with parameters such as the female sex, younger age, higher body mass index, higher anxiety score, higher prevalence of a history of cardiovascular disease, lower frequency of vigorous activities and more time spent sitting or reclining. These data may be of interest for assessing the burden of back and neck pain in Russia as part of the global burden of disease.

## Introduction

Low back pain and neck pain are among the most important causes of disability globally [1]. Low back and neck pain ranked in the list of the most frequent causes of global disability-adjusted life-years (DALYs) for both sexes; low back and neck pain were ranked 13th in 1990, 9th in 2006, and 4th in 2016 [1]. Among the 34 countries in the high-income regions worldwide, low back and neck pain were ranked 2nd in 1990, 2006 and 2016, while ischemic heart disease was the leading cause of DALYs. In previous studies, risk factors for back pain, thoracic spine pain and neck pain were old age, occupational hazards, a sedentary lifestyle, overweight, hereditary patterns, poor posture, pregnancy and smoking [2, 3]. To date, information about the prevalence of low back pain and neck pain in Russia and factors associated with the occurrence of low back and neck pain in Russia have been largely missing. In addition, information on the prevalence of low back pain, thoracic spine pain and neck pain have been relatively scarce for all other regions worldwide [2–16].

Since low back pain, thoracic spine pain and neck pain belong to the most common causes of the global burden of disease worldwide, and since information about their prevalence in Russia as the largest and one of the most populous countries worldwide is missing, we conducted this study to assess the prevalence of low back and neck pain in a population in Russia and to explore their associations with other major risk factors. In view of the multiethnic population in Russia, we additionally aimed to explore differences between an ethnic Russian population and a non-Russian population. The data would help to obtain information about the prevalence of neck pain, thoracic spine pain and low back pain in Russia with additional insight into interethnic differences. Data on the associations of neck, thoracic spine and low back pain with systemic and socioeconomic parameters would provide information about risk factors and potential preventive measures. Lastly, the data would increase the global knowledge on neck, thoracic spine and low back pain, which are public health-related problems, by adding information from an understudied area of the world. To assess the associations of neck pain, thoracic spine and low back pain with other factors in as much detail as possible, we included all parameters that were explored in the framework of the population-based study in the statistical analysis.

## Methods

In the study period from October 2015 to July 2017, we conducted the Ural Eye and Medical Study as a population-based study in the urban region of Kirovskii in Ufa, which is the capital of the republic of Bashkortostan, and in villages of the rural region of the Karmaskalinsky District at a distance of 65 km from Ufa [17–20]. The Ethics Committee of the Academic Council of the Ufa Eye Research Institute approved the study, and all participants gave informed written consent [20]. All people residing in the study regions were officially registered, and, according to the people registration, we performed home visits to inform the eligible individuals about the study and to invite them to participate. The home visits were repeated up to three times if the individuals were not met at home or needed some time to reflect about participating in the study. The only inclusion criteria for the study were living in the study region and an age of 40+ years, while there were no exclusion criteria.

We collected and reported data using the Guidelines for Accurate and Transparent Health Estimates Reporting: the GATHER statement guidelines [21]. Trained social workers carried out a standardized interview with more than 250 questions on socioeconomic parameters, diet, smoking or other types of tobacco consumption, daily physical activity, alcohol consumption, depression and suicidal thoughts, and medical history. The series of questions included the questions formulated in the Global Physical Activity Questionnaire by the World Health Organization [22]. In particular, the questionnaire included questions on the presence of neck pain, pain at the level of the thoracic spine, and low back pain, the known duration of the pain symptoms, and whether medication to reduce pain were taken. Using the definition of the International Association for the Study of Pain, neck pain was considered as pain perceived anywhere in the posterior region of the cervical spine, from the superior nuchal line to the first thoracic spinous process [23]. Thoracic spine pain was defined as pain located between the first thoracic spinous process and the 12th rib, and low back pain was defined as pain localized between the 12th rib and the inferior gluteal folds, with or without leg pain. The intensity of pain had to be at least so strong that it was perceived as “uncomfortable or worse than uncomfortable” and that it reduced the quality of life. The duration had to be at least 1 hour per day. The overall duration of pain was assessed as the number of months or years the pain had persisted. For all study participants, we assessed the arterial blood pressure, pulse rate, anthropomorphic parameters, handgrip strength and hearing loss [24, 25]. Blood samples taken under fasting conditions were biochemically examined. All participants underwent a pulmonary function test that included spirometric measurements [26]. We assessed depression using the Center for Epidemiologic Studies Depression Scale (CES-D), and we applied the State-Trait Anxiety Inventory (STAI) to assess trait and state anxiety.

In the statistical analysis, we calculated the prevalence of neck pain, thoracic spine pain and low back pain and presented the results as the means and 95% confidence intervals (CIs). In a univariate analysis, we searched for associations between the prevalence and severity of pain (back pain, neck pain or thoracic spine pain) and other parameters. The severity of pain was graded as whether or not analgesic tablets were taken. We finally conducted a multivariable binary regression analysis with the prevalence of pain as the dependent variable and as independent variables all those parameters that were significantly associated with pain in the univariate analysis. To assess potential interethnic differences between the Russian subgroup and the non-Russian subgroup, we stratified the whole study population and re-performed parts of the statistical analysis within the two subgroups. Odds ratios (ORs) and their 95% confidence intervals (CIs) were calculated. All *P-*values were two-sided and considered statistically significant when the values were less than 0.05.

## Results

Out of the 5889 individuals who were participating primarily in the Ural Eye and Medical Study, the present study included 5397 (91.5%) individuals with available information on the presence of neck pain, thoracic spine pain and low back pain. The mean age of the study population (2450 (45.4%) males) was 58.6 ± 10.6 years (median: 58 years; range: 40–94 years). The males were significantly younger than females (58.0 ± 10.3 years (range: 40–91 years) versus 59.1 ± 10.7 years (range: 40–94 years); *P* < 0.001). Using the results of the most recent Russian census carried out in 2010, our study population and the Russian population did not differ markedly in sex and age [27]. The mean body height was 165.0 ± 8.8 cm (median: 164 cm; range: 112–196 cm), the mean body weight was 75.9 ± 14.6 kg (median: 75 kg; range: 31–170 kg), and the mean body mass index was 27.9 ± 5.0 kg/m^2^ (median: 27.3 kg/m^2^; range: 13.96–60.96 kg/m^2^). The group of individuals with information on back pain as compared with the group of subjects without back pain data was significantly younger (58.6 ± 10.6 years versus 63.0 ± 11.3 years, *P* < 0.001), and had significantly more men (2450 (45.4%) men / 2947 (54.6%) women) versus 130 (25.9%) men / 372 (74.1%) women); *P* < 0.001).

The mean lifetime prevalence of low back pain was 2912 / 5397 (54.0%; 95% CI: 52.6, 55.3), and its mean duration was 12.7 ± 11.5 years. The mean lifetime prevalence and duration of thoracic spine pain were 1271 / 5397 (23.6%; 95% CI: 22.4, 24.7) and 11.8 ± 11.2 years, respectively, and the mean lifetime prevalence and duration of neck pain were 1570 / 5397 (29.1%; 95% CI: 27.9, 30.3) and 11.3 ± 10.9 years, respectively (Table 1). Among the 2912 individuals with low back pain, 703 (24.1%) took analgesic tablets.

**Table 1:** Prevalence (%) of low back pain, thoracic spine pain and neck pain in the Ural Eye and Medical Study, stratified by sex and age

Age Group (Years)	n	Low Back Pain	95% Confidence Intervals	Intake of Analgesic Tablets for Low Back Pain (Percentage)	95% Con-fidence Intervals	Thoracic Spine Pain	95% Con-fidence Intervals	Neck Pain	95% Con-fidence Intervals
Males									
40–44	209	45.6	38.7, 52.3	7.7	4.2, 11.0	13.9	9.2, 18.6	16.3	11.2, 21.3
45–49	350	46.9	41.6, 52.1	10.3	7.3, 13.0	14.9	11.1, 18.6	19.1	15.0, 23.3
50–54	422	50.2	45.5, 55.0	11.4	8.3, 14.3	19.7	15.9, 23.5	26.1	21.9, 30.3
55–59	471	47.8	43.2, 52.3	10.6	8.0, 13.0	19.7	16.1, 23.4	28.2	24.2, 32.3
60–64	383	50.4	45.4, 55.4	11.5	8.0, 15.0	17.8	13.9, 21.6	20.6	16.6, 24.7
65–69	273	46.2	40.2, 52.1	12.1	8.0, 16.0	13.9	9.8, 18.1	20.9	16.0, 25.7
70–74	124	44.4	35.5, 53.2	8.9	4.0, 14.0	14.5	8.2, 20.8	21.0	13.7, 28.2
75–79	150	50.0	41.9, 58.1	6.7	2.7, 10.8	14.0	8.4, 19.6	18.7	12.4, 24.5
80+	68	29.4	18.3, 40.5	2.9	−1.0, 7.0	5.9	0.1, 11.6	11.8	3.9, 19.6
Females									
40–44	252	55.6	49.4, 61.7	11.9	8.0, 16.0	25.0	19.6, 30.4	33.3	27.5, 39.2
45–49	361	59.6	54.5, 64.6	18.8	15.0, 23.0	28.0	23.3, 32.6	35.2	30.2, 40.1
50–54	465	60.9	56.4, 65.3	18.7	15.0, 22.0	30.1	25.9, 34.3	35.1	30.7, 39.4
55–59	500	57.6	53.3, 62.0	17.6	14.0, 21.0	28.2	24.2, 32.2	36.4	32.2, 40.6
60–64	449	63.9	59.5, 68.4	19.2	15.0, 23.0	34.5	30.1, 38.9	35.6	31.2, 40.1
65–69	430	62.8	58.2, 67.4	20.2	16.0, 24.0	31.6	27.2, 36.0	37.0	32.4, 41.6
70–74	182	56.6	49.3, 63.9	16.5	11.0, 22.0	29.1	22.5, 35.8	33.5	26.6, 40.4
75–79	203	53.7	46.8, 60.6	19.7	14.0, 25.0	24.1	18.2, 30.1	33.0	26.5, 39.5
80+	105	49.5	39.8, 59.3	19.0	11.0, 27.0	25.7	17.2, 34.32	23.8	15.5, 32.1

Within the Russian ethnic group (*n* = 1185; 508 men, 677 women) with a mean age of 60.1 ± 11.1 years, the prevalence and duration of neck pain was 372 / 1185 (31.4%; 95% CI: 28.8, 34.0) and 12.4 ± 11.9 years (range: 0–68 years), respectively, the prevalence and duration of thoracic spine pain was 313 / 1185 (26.4%; 95% CI: 23.9, 28.9) and 13.3 ± 12.5 years, respectively, and the prevalence and duration of low back pain was 685 / 1185 (57.8%; 95% CI: 55.0, 60.6) and 13.6 ± 12.0 years (range: 0.0–65.0 years), respectively. Comparing the Russian group with the non-Russian group revealed that the prevalence of neck pain (372 / 1185 (31.4%) versus 1198 / 4212 (28.4); *P* = 0.051; OR: 1.15; 95% CI: 1.00, 1.32), thoracic spine pain (313 / 1185 (26.4%) versus 958 / 4212 22.7%); *P* = 0.009; OR: 1.22; 95%: 1.05, 1.41), and low back pain (685 / 1185 (57.8%) versus 2227 / 42,412 (52.9%); *P* = 0.003; OR: 1.22; 95% CI: 1.07, 1.39) were higher in the Russian group. Similarly, the durations of thoracic spine pain (13.3 ± 12.5 years versus 11.3 ± 10.8 years; *P* = 0.008) and low back pain (13.6 ± 12.0 years versus 12.5 ± 11.3 years; *P* = 0.03) were significantly longer in the Russian group than in the non-Russian group, while the duration of neck pain (12.4 ± 11.9 years versus 10.9 ± 10.6 years; (*P* = 0.98) did not differ significantly between the Russian and the non-Russian group. In the non-Russian population, the different ethnicities (Bashkirs, Tartars, Chuvash, Mari and others) did not differ significantly (all *P* > 0.10) in the prevalence of neck pain, thoracic spine pain and lower back pain.

**Fig. 1:**
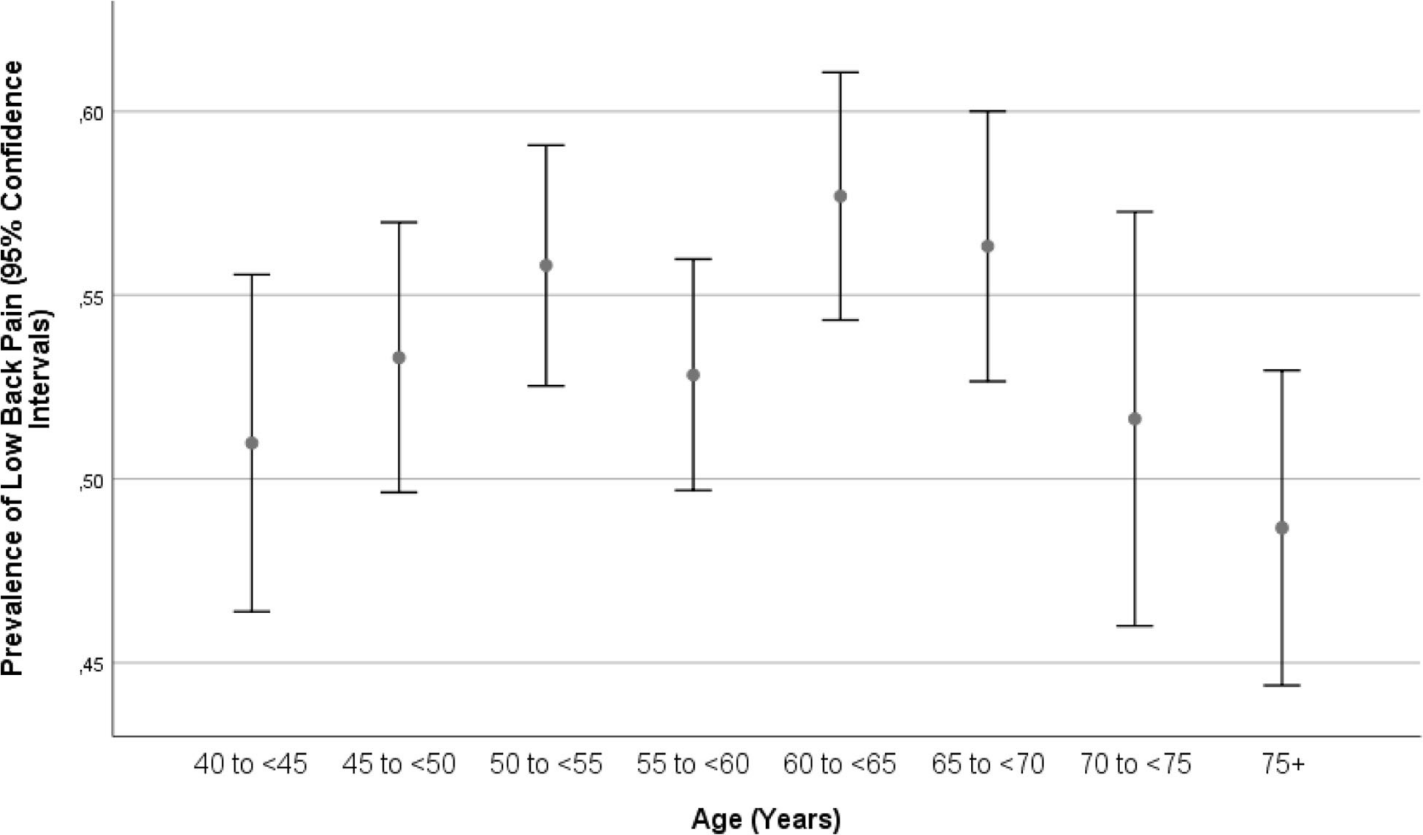
Graph showing the prevalence of low back pain stratified by age in the Ural Eye and Medical Study

In the univariate analysis, the prevalence of low back pain was correlated with female gender, anthropometric parameters including a higher body mass index, Russian ethnicity, parameters of physical activity and self-reported consumption of alcohol, smoking-related parameters, blood composition-related parameters such as higher blood concentrations of high-density lipoproteins, a higher prevalence of a history of arterial hypertension and other systemic diseases, younger age at the last menstruation, diet-related parameters, higher anxiety score and depression score, higher hearing loss score, lower dynamometric force and a higher prevalence of COPD, arterial hypertension and diabetes (Table 2). In univariate analysis, the prevalence of low back pain, thoracic spine pain and neck pain increased with age for individuals aged between 40 and 60 and decreased thereafter (Table 2) (Fig. 1). The subgroups with back pain (mean age: 58.5 ± 10.2 years), neck pain (58.5 ± 10.0 years) and thoracic spine pain (58.7 ± 9.9 years) did not differ significantly (*P* > 0.20) in age.

**Table 2:** Univariate associations between the prevalence of low back pain in the Ural Eye and Medical Study and other systemic parameters

Parameter	*P*-Value	Odds Ratio	95% Confidence Interval
Age (Years)	0.48	1.00	0.99, 1.00
Sex: Males / females	< 0.001	1.61	1.44, 1.79
Urban / rural region of habitation	0.07	0.90	0.81, 1.01
Family status: Married versus any other status	0.25	0.93	0.82, 1.05
Family type: Joint (three generations) / nuclear (two generations) / single / family of 2 people	0.27	1.03	0.98, 1.08
Ethnicity: Russian / any other ethnicity	0.003	1.22	1.07, 1.39
Body height (cm)	< 0.001	0.99	0.98, 0.99
Body weight (kg)	0.02	1.02	1.001, 1.008
Body mass index (kg/m^2^)	< 0.001	1.03	1.02, 1.04
Waist circumference (cm)	< 0.001	1.01	1.004, 1.012
Hip circumference cm)	< 0.001	1.02	1.01, 1.02
Waist-to-hip ratio	< 0.001	0.31	0.17, 0.55
Socioeconomic parameters			
Level of education (0–7)^a^	0.22	1.02	0.99, 1.05
Monthly income (below poverty line / average / above average / high)	0.03	0.88	0.79, 0.99
Own ownership of house (yes / no)	0.90	0.98	0.75, 1.29
Own ownership of refrigerator (yes / no)	0.08	0.32	0.09, 1.12
Own ownership of second house (yes / no)	0.17	1.13	0.95, 1.36
Own ownership of telephone (yes / no)	0.57	1.05	0.89, 1.23
Own ownership of smartphone (yes / no)	0.06	1.14	0.99, 1.31
Own ownership of television set (yes / no)	0.60	1.17	0.65, 2.12
Own ownership of car (yes / no)	0.19	1.11	0.95, 1.29
Own ownership of two-wheeler (yes / no)	0.35	0.95	0.85, 1.06
Own ownership of tractor (yes / no)	0.75	1.07	0.70, 1.64
Own ownership of bullock cart (yes / no)	0.35	1.25	0.78, 1.99
Own ownership of computer / laptop (yes / no)	0.29	1.08	0.93, 1.25
Physical activity			
How long is your usual workday? (minutes)	0.20	1.00	1.00, 1.00
Does your work involve mostly sitting or standing with less than 10 min of walking at a time? (yes / no)	0.28	0.93	0.82, 1.06
Does your work involve physically vigorous activity (such as heavy lifting or digging) or physically moderate intensity activity (such as brisk walking or carrying light loads) (yes / no)	0.03	0.88	0.78, 0.99
How many days a week do you do such physically vigorous activity during work? (number of days)	0.11	0.96	0.92, 1.01
On a usual day, how much time do you spend on such physically vigorous work during work? (minutes)	0.51	1.00	1.00, 1.00
Does your work involve physically moderate-intensive activity, such as brisk walking or carrying light loads, for at least 10 min at a time? (yes / no)	0.97	1.00	0.88, 1.13
On average, including all days of the week, how much time do you spend performing physically moderate to intensive activities as part of your work each day? (minutes)	0.54	1.00	1.00, 1.00
In a typical week, on how many days do you do physically moderate activities as part of your work? (number of days)	0.08	0.96	0.92, 1.01
Do you walk or use a bicycle (pedal cycle) for at least 10 min continuously to get to and from places? (yes / no)	0.002	0.74	0.61, 0.99
In a typical week, on how many days do you walk or use a bicycle (pedal cycle) for at least 10 min continuously to get to and from places? (number of days)	0.04	0.96	0.92, 0.998
How much time do you spend walking or bicycling for travel in a day? (minutes)	0.98	1.00	1.00, 1.00
Does your recreation, sport or leisure time involve mostly sitting, reclining or standing activities, with no physical activity lasting more than 10 min at a time? (yes / no)	0.73	1.02	0.91, 1.14
In your leisure time, do you do any physically vigorous activities such as running, strenuous sports or weightlifting for at least 10 min at a time? (yes / no)	< 0.001	0.74	0.65, 0.83
If yes, in a typical week, on how many days do you do physically vigorous activities as part of your leisure time? (number of days)	0.36	0.98	0.93, 1.03
How much time do you spend on physically vigorous activities as part of your leisure time on a typical day? (minutes)	0.70	1.00	0.999, 1.001
In a typical week, on how many days do you do physically moderately intensive activities such as brisk walking, cycling or swimming for at least 10 min at a time in your leisure time? (number of days)	0.01	0.96	0.92, 0.99
In a typical week, on how many days do you do physically moderate to intensive activities as part of your leisure time? (number of days)	0.01	0.96	0.92, 0.99
How much time do you spend on physically moderate to intensive activities per day during a week in your leisure time? (minutes)	0.003	0.999	0.999, 1.000
Over the past 7 days, how much time did you spend sitting or reclining in a typical day? (minutes)	0.007	1.00	1.00, 1.00
History of disease			
History of angina pectoris (yes / no)	< 0.001	2.67	2.15, 3.29
History of asthma (yes / no)	0.008	1.61	1.13, 2.28
History of arterial hypertension (yes / no)	< 0.001	1.38	1.24, 1.55
History of arthritis (yes / no)	< 0.001	2.43	2.14, 2.77
History of bone fracture (yes / no)	< 0.001	1.28	1.13, 1.43
History of headache (yes / no)	< 0.001	2.73	2.44, 3.05
History of cancer (yes / no)	0.87	1.03	0.74, 1.42
History of a cardiovascular disorder including stroke (yes / no)	< 0.001	1.65	1.46, 1.87
History of dementia (yes / no)	0.19	1.58	0.80, 3.11
History of diabetes mellitus (yes / no)	0.02	1.26	1.04, 1.54
History of diarrhea (yes / no)	0.003	4.94	1.71, 14.3
History of iron-deficiency anemia (yes / no)	< 0.001	2.69	2.07, 3.51
History of low blood pressure and hospital admittance (yes / no)	< 0.001	1.92	1.39, 2.64
History of osteoarthritis (yes / no)	< 0.001	2.95	2.53, 3.44
History of skin disease (yes / no)	< 0.001	1.72	1.34, 2.21
History of thyroid disorder (yes / no)	< 0.001	1.92	1.59, 2.33
History of falls (yes / no)	< 0.001	2.08	1.80, 2.40
History of unconsciousness (yes / no)	< 0.001	2.37	1.91, 2.94
Age at the last menstruation (years) (yes / no)	0.03	0.98	0.97, 0.998
Age at the last regular menstruation (years) (yes / no)	0.01	1.98	0.96, 0.995
History of menopause (yes / no)	0.006	1.29	1.08, 1.56
Blood concentrations (mmol/L) of:			
Alanine aminotransferase (IU/L)	0.62	0.999	0.995, 1.003
Aspartate aminotransferase (IU/L)	0.47	0.998	0.993, 1.003
Bilirubin, total (μmol/L)	0.89	1.00	0.996, 1.005
High-density lipoproteins (mmol/L)	0.002	1.10	1.04, 1.17
Low-density lipoproteins (mmol/L)	0.92	1.002	0.958, 1.05
Triglycerides (mmol/L)	0.06	1.07	0.998, 1.16
Cholesterol (mmol/L)	0.01	1.04	1.01, 1.08
C-reactive protein (mg/L)	0.20	1.13	0.94, 1.36
Rheumatoid factor (IU/mL)	0.64	0.99	0.93, 1.05
Erythrocyte sedimentation rate (mm / hour)	0.24	1.00	0.998, 1.008
Glucose (mmol/L)	0.21	1.02	0.99, 1.06
Prevalence of diabetes mellitus	0.03	1.21	1.02, 1.44
Creatinine (μmol/L)	< 0.001	0.97	0.993, 0.998
Urea (mmol/L)	0.84	1.00	0.96, 1.03
Residual nitrogen (g/L)	0.86	0.94	0.45, 1.94
Total protein (g/L)	0.44	1.00	0.995, 1.01
International normalized ratio (INR)	0.001	0.53	0.36, 0.76
Blood coagulation time	0.04	1.11	1.003, 1.23
Prothrombin time (%)	< 0.001	1.01	1.01, 1.02
Hemoglobin	0.02	0.996	0.992, 0.999
Erythrocytes (10^6^ cells / μL)	0.01	0.83	0.72, 0.96
Leukocytes (10^9^ cells / L)	0.19	0.98	0.94, 1.01
Rod-core granulocyte (% of leukocytes)	0.51	1.01	0.97, 1.06
Segment nuclear granulocyte (% of leukocytes)	0.61	1.00	0.99, 1.01
Eosinophil granulocytes (% of leukocytes)	0.047	1.06	1.001, 1.13
Lymphocytes (% of leukocytes)	0.93	1.00	0.99, 1.01
Monocytes (% of leukocytes)	0.60	0.99	0.97, 1.02
Blood pressure, systolic (mmHg)	0.50	1.00	0.996, 1.002
Blood pressure, diastolic (mmHg)	0.13	1.00	0.999, 1.01
Blood pressure, mean (mmHg)	0.77	1.00	0.995, 1.01
Prevalence of arterial hypertension	0.005	1.17	1.05, 1.30
Prevalence of chronic obstructive pulmonary disease	< 0.001	1.58	1.27, 1.97
Diet			
Vegetarian diet / mixed diet	0.33	2.35	0.43, 12.8
Number of meals per day	0.97	1.00	0.93, 1.07
In a week how many days do you eat fruits? (number of days)	< 0.001	0.93	0.91, 0.96
How many servings of fruit do you eat on one of those days (g)	0.82	1.00	1.000, 1.001
In a week how many days do you eat vegetables?	0.82	1.00	1.00, 1.01
How many servings of vegetables do you eat on one of those days (g)?	0.001	1.001	1.000, 1.001
Type of oil used for cooking: vegetable oil / non-vegetable oil	0.06	1.22	0.99, 1.55
Foods containing whole grains (yes / no)	0.07	0.89	0.78, 1.01
Salt consumed per day (g)	0.001	0.96	0.94, 0.99
Degree of processing meat (weak / medium / well done)	0.70	1.02	0.92, 1.13
Smoking			
Do you currently smoke any tobacco products? (yes / no)	< 0.001	0.75	0.64, 0.88
Do you smoke daily? (yes / no)	0.001	0.76	0.64, 0.89
Number of packages per year (package = 20 cigarettes)	0.03	0.995	0.991, 1.000
Alcohol			
Do you consume alcohol such as beer, whisky, rum, gin brandy or other local products? (yes / no)	0.003	0.83	0.73, 0.94
How many alcoholic drinks do you have on a typical day when you are drinking? (number of drinks)	0.001	0.999	0.998, 1.000
How often do you have 6 or more drinks on one occasion? (never / rarely / sometimes / often / cannot say)	0.09	1.14	0.98, 1.33
Hearing loss			
Hearing loss total score	0.002	1.01	1.003, 1.01
Depression			
Depression score	< 0.001	1.07	1.06, 1.09
State-Trait Anxiety Inventory (STAI)			
Anxiety score	< 0.001	1.08	1.07, 1.10
Dynamometry			
Manual dynamometry, right hand (dekaNewtons)	< 0.001	0.99	0.98, 0.99
Manual dynamometry, left hand (dekaNewtons)	< 0.001	0.99	0.98, 0.99

^a^The level of education was categorized into the stages of “illiteracy” (no reading ability at all), “passing of the 5th class”, “passing of the 8th class”, “passing of the 10th class”, “passing of the 11th class”, “graduation”, and “post-graduation”

In the binary regression analysis with the occurrence of low back pain as dependent variable, we excluded due to collinearity the parameters of body weight, hip circumference and frequency of smoking within a day. Due to a lack of statistical significance, we then sequentially excluded the predictors that were no longer significantly associated with the outcome parameter. In the resulting final model, a higher prevalence of lower back pain was associated with females, younger age, higher body mass index, lower frequency of vigorous activities during leisure time, more time spent sitting or reclining during the last 7 days, higher blood concentration of high-density lipoproteins, higher prothrombin index, higher prevalence of a history of cardiovascular disease, falls, unconsciousness, bone fractures, osteoarthritis, iron-deficiency anemia and thyroid disorder, lower amount of salt consumption, fewer days with vegetable intake, and a higher anxiety score (Table 3). If the history of menopause was added to the model in women, it was significantly associated with the prevalence of low back pain (*P* = 0.02; OR: 1.36; 95% CI: 1.05, 1.75), while the prothrombin index (*P* = 0.14), the history of bone fracture (*P* = 0.08) and the amount of salt consumed (*P* = 0.08) were no longer significantly associated with the prevalence of low back pain.

**Table 3:** Associations (multivariate analysis) of the prevalence of low back pain in the Ural Eye and Medical Study

Parameter	*P*-Value	Odds Ratio	95% Confidence Interval
Age (Years)	< 0.001	0.99	0.98, 0.99
Sex (Males / Females)	0.04	1.14	1.004, 1.30
Body Mass Index (kg/m^2^)	0.002	1.02	1.01, 1.03
Vigorous Activity during Leisure Time (Minutes)	0.001	0.79	0.69, 0.90
Time Spent Sitting or Reclining during the Last 7 Days (Minutes)	0.03	1.00	1.00, 100
Blood Concentration of High-Density Lipoproteins (mmol/L)	0.004	1.10	1.03, 1.18
Prothrombin Index Concentration	0.003	1.01	1.003, 1.01
History of Cardiovascular Disease (Yes / No)	0.004	1.23	1.07, 1.42
History of Falls (Yes / No)	< 0.001	1.71	1.45, 2.00
History of Unconsciousness (Yes / No)	< 0.001	1.78	1.40, 2.25
History of Bone Fracture (yes / no)	0.01	1.18	1.03, 1.34
History of Osteoarthritis (Yes / No)	< 0.001	2.76	2.34, 3.26
History of Iron-Deficiency Anemia (Yes / No)	< 0.001	1.87	1.41, 2.50
History of Thyroid Disorder ((Yes / No)	0.004	1.37	1.10, 1.70
Number of Days with Intake of Vegetables per Week (number of days)	< 0.001	0.89	0.85, 0.93
Salt Intake (g)	0.008	0.97	0.94, 0.99
Anxiety Score	< 0.001	1.05	1.03, 1.06

The intake of analgesic tablets for low back pain was associated with a younger age, females, more time spent sitting or reclining during the last 7 days, lower prevalence of vigorous activities during leisure time, higher prevalence of a history of cardiovascular disease, falls, unconsciousness and osteoarthritis, and higher anxiety score (Table 4). In that model, body mass index (*P* = 0.41) and prevalence of iron deficiency anemia (*P* = 0.82) and thyroid disorder (*P* = 0.09) were not significantly associated with the intake of analgesic tablets.

**Table 4:** Associations (multivariate analysis) of the prevalence of intake of analgesic tablets for low back pain in the Ural Eye and Medical Study

Parameter	*P*-Value	Odds Ratio	95% Confidence Interval
Age (Years)	0.002	0.99	0.98, 0.99
Sex (Males / Females)	< 0.001	1.45	1.21, 1.75
Vigorous Activity during Leisure Time (No / Yes)	< 0.001	0.65	0.52, 0.80
Time Spent Sitting or Reclining during the Last 7 Days (Minutes)	< 0.001	1.00	1.00, 1.00
History of Cardiovascular Disease (Yes / No)	< 0.001	1.47	1.22, 1.78
History of Falls (Yes / No)	0.001	1.40	1.15, 1.71
History of Unconsciousness (Yes / No)	0.04	1.33	1.01, 1.75
History of Osteoarthritis (Yes / No)	< 0.001	2.76	2.76, 3.35
Anxiety Score	< 0.001	1.08	1.06, 1.11

A higher prevalence of thoracic spine pain was significantly associated with female gender, higher body mass index, higher prevalence of a history of cardiovascular disease, falls, unconsciousness, bone fractures, osteoarthritis, iron-deficiency anemia and thyroid disorder, and higher anxiety score (Table 5).

**Table 5:** Associations (multivariate analysis) of the prevalence of thoracic spine pain in the Ural Eye and Medical Study

Parameter	*P*-Value	Odds Ratio	95% Confidence Interval
Age (Years)	< 0.001	0.99	0.98, 0.99
Sex (Males / Females)	< 0.001	1.42	1.22, 1.67
Body Mass Index (kg/m^2^)	0.03	1.02	1.002, 1.03
History of Cardiovascular Disease (Yes / No)	< 0.001	1.71	1.47, 1.99
History of Falls (Yes / No)	< 0.001	1.46	1.24, 1.72
History of Unconsciousness (Yes / No)	< 0.001	1.69	1.35, 2.11
History of Bone Fracture (Yes / No)	0.01	1.18	1.03, 1.34
History of Osteoarthritis (Yes / No)	< 0.001	2.58	2.21, 3.01
History of Iron-Deficiency Anemia (Yes / No)	0.002	1.52	1.17, 1.98
History of Thyroid disorder (Yes / No)	0.01	1.32	1.07, 1.63
Anxiety Score	< 0.001	1.09	1.07, 1.11

A higher prevalence of neck pain was significantly associated with females, a younger age (Fig. 3), a higher body mass index, a higher prevalence of vigorous activity during leisure time, a higher amount of time spent sitting, and a higher prevalence of a history of cardiovascular disease, falls, unconsciousness, osteoarthritis, iron-deficiency anemia and thyroid disorder, and a higher anxiety score (Table 6).

**Table 6:** Associations (multivariate analysis) of the prevalence of neck pain in the Ural Eye and Medical Study

Parameter	*P*-Value	Odds Ratio	95% Confidence Interval
Age (Years)	< 0.001	0.99	0.98, 0.99
Sex (Males / Females)	< 0.001	1.33	1.16, 1.53
Body Mass Index (kg/m^2^)	0.02	0.98	0.97, 0.997
Vigorous Activity during Leisure Time (Yes / No)	0.002	0.79	0.69. 0.92
Time Spent on Sitting or Reclining during the Last 7 Days (Minutes)	0.006	1.00	1.00, 1.00
History of Cardiovascular Disease (Yes / No)	< 0.001	1.51	1.30, 1.74
History of Falls (Yes / No)	< 0.001	1.62	1.39, 1.90
History of Unconsciousness (Yes / No)	< 0.001	1.65	1.33, 2.05
History of Osteoarthritis (Yes / No)	< 0.001	3.06	2.63, 3.57
History of Iron-Deficiency Anemia (Yes / No)	0.001	1.56	1.21, 2.02
History of Thyroid disorder (Yes / No)	0.003	1.36	1.11, 1.68
Anxiety Score	< 0.001	1.06	1.04, 1.08

## Discussion

The findings of our study suggest that the mean prevalence of low back pain, thoracic spine pain and neck pain in our study population from Russia was 54.0, 23.6 and 29.1%, respectively. A higher prevalence of lower back/neck/thoracic spine pain was associated with female gender, younger age and a variety of parameters including higher body mass index, lower frequency of vigorous activities during leisure time, more time spent sitting and reclining, higher prevalence of a history of cardiovascular disease, falls, bone fractures, unconsciousness, osteoarthritis, iron-deficiency anemia and thyroid disorder, lower vegetable intake, and a higher anxiety score.

The findings obtained in our study agree with observations made in previous investigations on other ethnic groups. The lifetime prevalence of low back pain was 54% in our study population of individuals aged 40+ years, which was higher than the prevalence reported in previous investigations of other study populations in other countries [2–16]. Urwin and colleagues estimated the frequency of musculoskeletal pain in different anatomical sites in an adult population in general medical practices in the Greater Manchester region in the UK [6]. After an adjustment was made for social deprivation, the most common site of pain was the back (prevalence: 23% (95% CI: 21, 25)) followed by the knee (19% (95% CI: 18, 21)) and shoulder (16% (95% CI: 14, 17)). Johansson and colleagues performed a systematic literature review to assess the frequency of mid-back pain (or thoracic spine pain) [3]. Summarizing seven studies, they reported that the 3-month and 2-year incidence rates of mid-back pain in children and adolescents was 4 and 50%, respectively. In adults, the 1-month incidence rate was less than 1%. After performing a literature review, Meucci and coworkers estimated the global prevalence of chronic low back pain [14]. The prevalence of chronic low back pain was 4.2% in individuals aged between 24 and 39 and 19.6% in those aged between 20 years and 59 years. The authors also reported that chronic low back pain prevalence increased linearly from the third decade of life until the age of 60 years and that it was more prevalent in women. Jackson and colleagues performed a meta-analysis of 119 studies in 28 low-to-medium income countries and reported an average prevalence of low back pain of 28% (95% CI: 16, 42) [15]. Garcia performed a systematic review of chronic nonspecific low back pain in Latin America and found a mean prevalence of chronic low back pain of 31.3% [13]. Some of the reasons for the discrepancies in the prevalence of low back pain between the various studies may be the differences between the study populations in terms of age, the time of the study, the inclusion of low-income countries versus high-income countries, the type of work, the differences in the definition of low back pain, and others. All studies reported a relatively high prevalence of low back pain in the general adult and elderly population and underscore the importance of low back pain as one of the leading level 3 causes of total DALYs in the Global Burden of Disease Study [1].

Additionally, the prevalence of neck pain was 29% in our study population, which was higher than the prevalence reported in other studies. Noormohammadpour and colleagues carried out a cross-sectional, population-based survey of 7889 Iranians aged 30 to 70 and found a prevalence of chronic neck pain and chronic low back pain of 15.3 and 27.2%, respectively [28]. In Noormohammadpour’s study, a higher prevalence of neck pain was associated with females, an older age, a body mass index ≥25 kg/m^2^, a lower level of education, passive smoking, a history of osteoporosis, and low or high physical activity levels. In a population-based study of 34,902 Danish twins with an age of 20–71 years by Leboeuf-Yde and associates, 12, 10, and 4% of the study participants reported having back pain, neck pain and thoracic pain, respectively, for at least 30 days in the previous year [29]. In the framework of the MONICA health survey, Guez and associates selected 4415 individuals with an age of 25–64 years and found a prevalence of chronic low back pain and chronic neck pain of 16 and 17%, respectively, with 51% of subjects having both back pain and neck pain [ 30]. Genebra and colleagues reported a prevalence of neck pain of 20.3% in 600 Brazilian individuals [31]. A higher prevalence of neck pain was correlated with the family status of being a widow and being separated, a low educational level or low income, and mostly sitting during work. All studies reproted that low back pain occurred more commonly than neck pain and that thoracic spine pain was the least common.

The prevalence of thoracic spine pain in our study population was 29.1% (95% CI: 27.9, 30.3). In a study by Fouquet N and coworkers on a group of 3710 workers (58% men) aged 20–59 years, the prevalence of thoracic spine pain in a week was 17% in females and 9% in males [32]. The prevalence was higher in lower-class, male, white-collar workers than in male workers in other occupational categories, and it was higher in upper-class, female, white-collar workers than in professional workers. After performing a cross-sectional study on 34,902 twin individuals with an age of 20 to 71 years, Leboeuf-Yde and colleagues found that pain with a duration of ≥30 days within the last year was reported by 4% of the study participants [29]. Females, compared to males, had a higher prevalence of thoracic spine pain. As in our study, the prevalence of thoracic spine pain increased with age up to an age of approximately 55 to 60 years and then decreased.

**Fig. 2:**
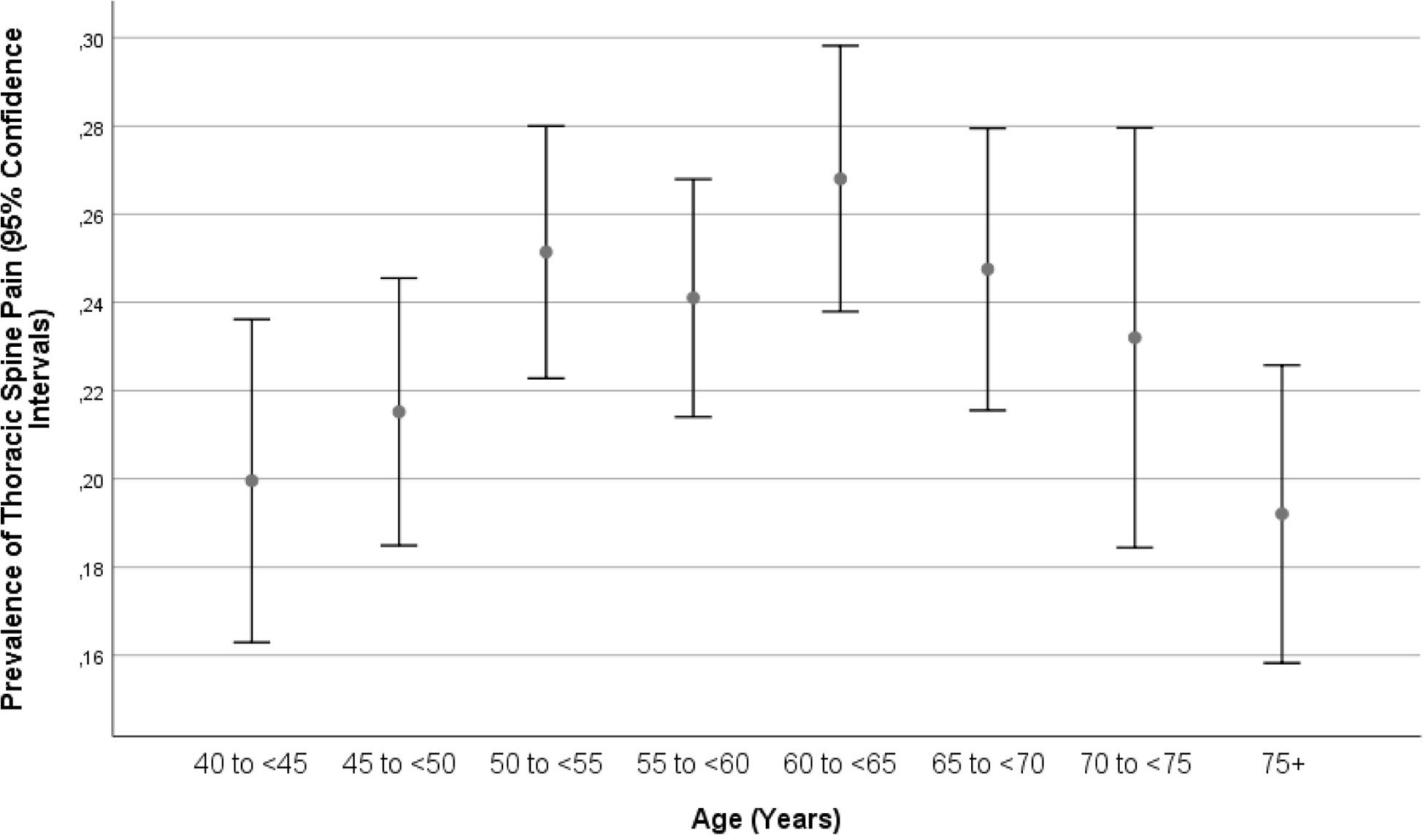
Graph showing the prevalence of thoracic spine pain stratified by age in the Ural Eye and Medical Study

**Fig. 3:**
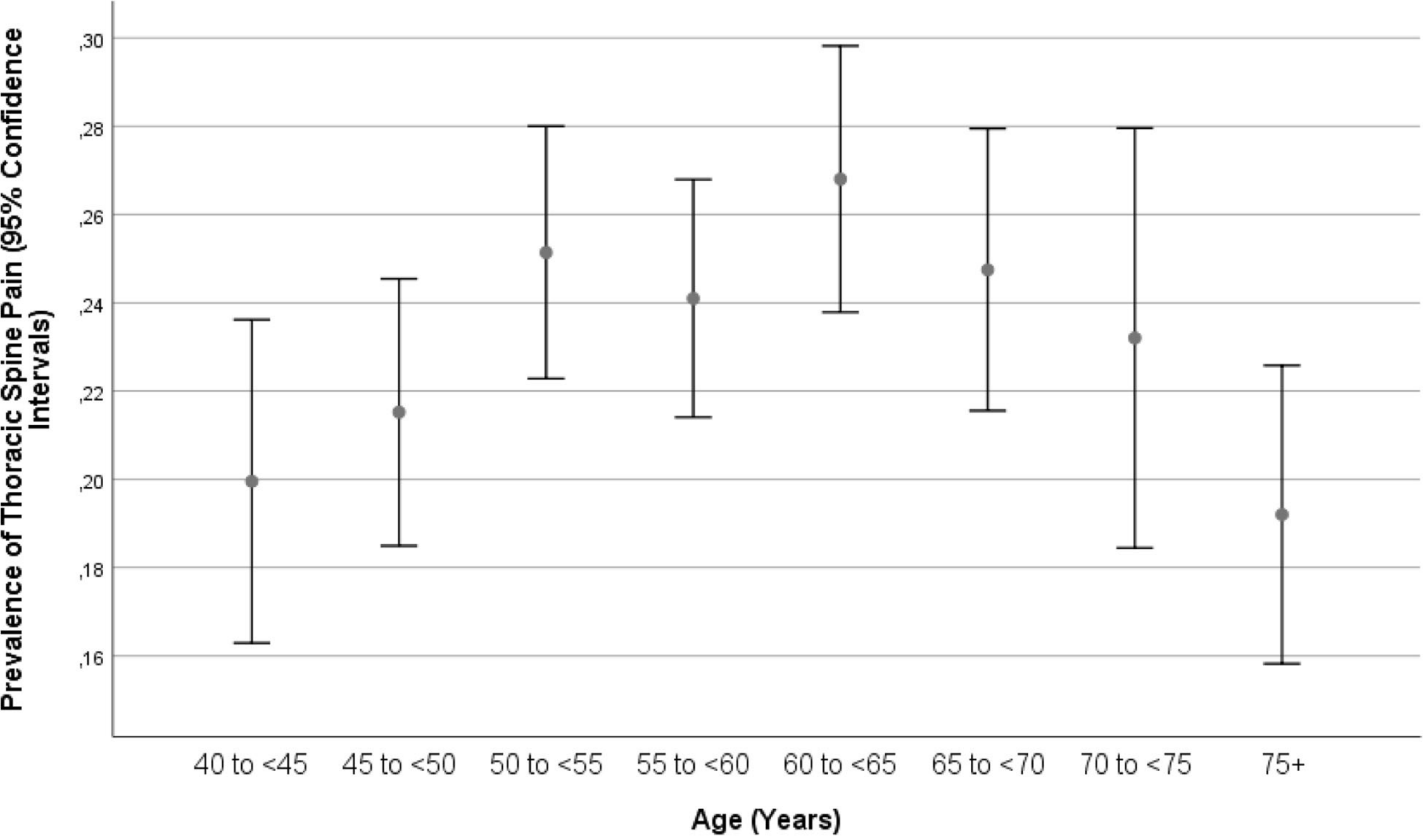
Graph showing the prevalence of neck pain stratified by age in the Ural Eye and Medical Study

The reasons for discrepancies in the prevalences of low back pain, thoracic spine pain and neck pain between our studies and preceding investigations may include the differences in the study population, differences in the definition of the disorders, differences in the prevalences of risk factors for back pain, thoracic spine pain and neck pain, and others. All studies reported that the prevalence of low back pain was higher than the prevalence of the other two pain-associated disorders. In our study, the prevalence of thoracic spine pain was higher than the prevalence of neck pain, while in other studies, neck pain was more common. In many studies, as in our investigation, the prevalence of the pain disorders increased with age up to an age of approximately 60 years and then decreased (Figs. 1, 2, and 3). This nonlinear, inverted U-like shape of the association between pain prevalence and age may have been the reason for the discrepancy between some studies in which the prevalence of pain disorders increased or decreased with age.

A higher prevalence of low back pain, thoracic spine pain and neck pain were correlated with various factors, including females, a higher body mass index, a higher prevalence of a history of osteoarthritis, a higher anxiety score, a lower frequency of vigorous activities during leisure time and more time spent sitting or reclining during the last 7 days (Tables 2-5). Similar associations were reported in previous studies [33–38]. The associations between the prevalence of low back pain, thoracic spine pain and neck pain with age differed between the studies, probably due to the nonlinear relationship with an inverted U-shape curve (Figs. 1, 2, and 3) [34]. In contrast to our study, Shiri and colleagues found, a modest association (OR 1.33; 95% CI: 1.26–1.41) between both current and former frequencies of smoking and a higher prevalence of low back pain [39]. The association between a higher prevalence of the pain disorders and a higher body mass index might be caused by a larger load on the vertebral column due to a larger body weight [40]. The higher prevalence of pain disorders in females compared to males might have been associated with osteoporosis, which is generally more common in females than in males. Additionally, differences in the lifestyle between males and females might have played a role. The association with a higher anxiety score may have been a result of increases in insecurity and fear due to the pain. The correlation between a lower prevalence of back pain and a higher frequency of vigorous activities during leisure may be due to a more active lifestyle strengthening the muscles and reducing the risk for back pain. As a corollary, more time spent sitting or reclining was associated with a higher prevalence of pain-associated disorders. In a parallel manner, the correlation between a higher prevalence of back/neck/thoracic spine pain and a history of cardiovascular diseases may be explained by the association between a more sedentary lifestyle, back pain and cardiovascular problems. Also, the relationship between a higher prevalence of back/neck/thoracic spine pain and lower intake of vegetables may belong to the same circle of parameters on unhealthy lifestyle and back/neck/thoracic spine pain. The association between a higher prevalence of back/neck/thoracic spine pain and a higher prevalence of previous falls and bone fractures might have been bi-directional, with previous bone fractures potentially leading to back/neck/thoracic spine pain.

When the findings of our study are discussed, its limitations should be mentioned. First, although not a specific limitation of the present study, the definition of low back pain, thoracic spine pain and neck pain varied between previous investigations, so the results of the various studies cannot easily be compared. To cite an example, some of the previous studies used a definition of spinal pain with a duration of 30 days within the last year, while for our study, the intensity of pain had to be at least so strong that it was perceived as “uncomfortable or worse than uncomfortable”, that it reduced the quality of life, and that its duration had to be at least 1 hour per day. These variations in the definition of pain will have caused differences in the results when different studies are compared. Second, we attempted to grade the severity of pain by the information whether or not analgesic tablets were taken (Table 4). One may have to take into account that the use of analgesics depends on a variety of factors independently of the pain itself and which include the individual tolerability of pain and the access to the analgesic medication, to mention only a few. Third, one has to take into account in the discussion about the relationships between the prevalence of back/neck/thoracic spine pain and other parameters, that the study design did not allow any assessment about the causes of these relationships. In particular, one has to consider that these relationship could be going in either direction and many of the associations could be a result of the back/neck/thoracic spine pain rather than the preceding cause of the pain. The strengths of the study are that it is its first of its size and design for Russia and Eastern Europe, that it addressed not only back pain but a multitude of other parameters and diseases, which allowed the exploration of potentially hidden associations between back pain and these diseases, and that it recruited study participants from a rural region and an urban region.

## Conclusions

In conclusion, in this common, ethically mixed, urban and rural Russian population aged 40+ years, the prevalence of low back pain, thoracic spine pain and neck pain were 54.0, 23.6 and 29.1%, respectively. The associations of the prevalence of low back pain, thoracic spine pain and neck pain with age were nonlinear, showing an inverted U-shape curve with the climax at an age of 60 years. A higher prevalence of low back pain, thoracic pain and neck pain were associated with females, a higher body mass index, a higher prevalence of a history of cardiovascular disease, falls, unconsciousness, osteoarthritis, iron-deficiency anemia and thyroid disorder, and a higher anxiety score. The prevalence of low back pain and neck pain was also correlated with a lower frequency of vigorous activities during leisure time and more time spent sitting or reclining during the last 7 days. The data may be of interest for assessing the burden of back and neck pain in Russia as part of the global burden of disease.

## Data Availability

Available upon request from the corresponding author.

## Abbreviations

BMI: Body mass index
CI: Confidence interval
DALYs: Disability-adjusted life-years
FEV1: Forced expiratory volume in one second
Fig: Figure
FVC: Forced vital capacity
GATHER: Guidelines for Accurate and Transparent Health Estimates Reporting
OR: Odds ratio
SPSS: Statistical Package for Social Science
